# Recyclability of Asphalt Mixtures with Crumb Rubber Incorporated by Dry Process: A Laboratory Investigation

**DOI:** 10.3390/ma13122870

**Published:** 2020-06-26

**Authors:** Israel Rodríguez-Fernández, Maria Chiara Cavalli, Lily Poulikakos, Moises Bueno

**Affiliations:** 1 EMPA, Swiss Federal Laboratories for Material Science and Technology, 8600 Dübendorf, Switzerland; israel.rodriguezf@gmail.com (I.R.-F.); mcca@kth.se (M.C.C.); Lily.Poulikakos@empa.ch (L.P.); 2 GITECO Research Group, University of Cantabria, 39005 Santander, Spain; 3 Structural Engineering and Bridges, School of Architecture and the Built Environment, KTH Royal Institute of Technology, 11428 Stockholm, Sweden

**Keywords:** asphalt, crumb rubber, dry process, aging, recyclability

## Abstract

Semi-Dense Asphalt (SDA) mixtures are nowadays recommended for the surface layer of low noise roads in urban areas due to their optimal functional characteristics. Moreover, the use of polymer-modified bitumen (PmB) in its design results in high mechanical performance. However, this type of highly modified bitumen implies significant economic and environmental disadvantages. The polymer modification increases the production cost, involves higher mixing temperatures, and makes the recycling process of the asphalt mixtures challenging. As a potential alternative to PmB in SDA mixtures, this experimental work analyses the dry process for the incorporation of crumb rubber (CR) from waste tires. Particularly, the main objective was to study the aging effect and the recyclability of asphalt mixtures prepared in the laboratory with two different types of CR. The volumetric properties and mechanical performance of the mixtures artificially aged and rejuvenated were evaluated. The results obtained show that mixtures with CR have adequate performance, being less susceptible to aging than a conventional polymer-modified mixture. Furthermore, the rheological response of asphalt binder samples recovered from the mixtures at different aging states was analyzed. It was observed that the effect of the rejuvenator depended on the CR type, but this fact did not negatively influence the performance of the recycled mixtures.

## 1. Introduction

The road network plays a very important role in the transport of people and goods as roads are the most widely used mode of transport. Recent statistics on freight and passenger transport show that more than 70% of goods and 80% of passengers travel by road. More than 90% of the European road network is surfaced with asphalt, providing road users a consistent, safe, and durable surface for their journeys, whether local or across the entire continent [1].

In recent years, new designs of asphalt mixtures have allowed the road surface quality to be improved. As an example, Semi-Dense Asphalt (SDA) mixtures combine high mechanical performance with other functional characteristics, such as the reduction of the noise from the tire/pavement interaction, making SDA especially interesting for urban areas [2]. Due to their design (10–20% air void content), this type of mixture requires the use of polymer-modified bitumen (PmB). It is widely known that, in comparison with virgin bitumen, these highly modified bitumens offer higher stiffness at high temperatures, higher cracking resistance at low temperatures, better moisture resistance, and longer fatigue life [3,4]. However, they have major disadvantages that involve their high cost, high mixing temperature, high temperature sensitivity, low aging resistance, poor storage stability, and the limited improvement in elasticity [5].

The use of rubber from waste tires as elastic additive in asphalt mixtures has become a realistic alternative to asphalt mixtures with PmB for high performance roads. In addition, this implies significant environmental benefits. Along with the direct reduction of end of life tires, the feasibility of the rubberized asphalt for production processes with low energy consumption, as well as the decrease of emissions (CO and methane) for conventional hot asphalt production processes, have been reported lately [6,7,8]. Furthermore, it is shown that an optimal incorporation of crumb rubber (CR) particles as elastic aggregate can manage to reduce the noise from the tire/pavement interaction up to 2 dB(A) [9]. All these factors allow considering the use of CR as a potential candidate for SDA mixtures.

In order to obtain a suitable rubber for asphalt mixture production, waste tires must be followed a recovery process called “granulate recovery”. In this process, the waste tires are first cut into small pieces and the fabrics, as well as metallic components, are removed. Then, the size of the rubber is further reduced, resulting in Crumb Rubber (CR). Ambient grinding, cryogenic grinding, wet-grinding, and hydro jet size reduction are some of the technologies most commonly used. The final size and shape of the resultant CR particles will vary depending on the process employed [8,10].

The technique that incorporates the CR into the asphalt mixtures as part of the aggregates is commonly known as the dry process [11]. During the early years of this technology, different practical attempts ended facing common problems associated with a poor reproducibility or premature failures of road surfacing [12,13,14]. Primarily, the poor interaction between the CR and the asphalt binder was defined as the main cause of the irregular performance of CR modified mixtures. This directly affected the cohesion within the mixture that finally led to a reduction in the bearing capacity, raveling, and lower resistance to moisture of the pavement. These initial limitations resulted in a certain lack of confidence in the dry process, delaying its development [15]. Moreover, it was seen that key manufacturing parameters, such as the temperature and mixing time, had an important influence, as well [15]. In particular, after the mixing process, a specific conditioning time at relatively high temperature was usually required to ensure a proper interaction between the CR and the binder (partial swelling). Today, many research and practical works have confirmed that this conditioning time has a major effect on short-term performance of the modified mixtures [15,16].

In the last few decades, the methodology that guarantees a better performance of CR modified mixtures has been developed [17]. Along with it, different treatments have been studied to chemically activate the rubber particles with the aim to enhance their interaction within the asphalt matrix [15,17,18,19,20,21,22,23,24]. The current rubber solutions can now offer a double function in the behavior of the asphalt mixture. On the one hand, it acts as an elastic aggregate, and, on the other hand, it can partially modify the properties of the binder. These two functions have been found to improve the resistance to cracking at low temperature, permanent deformation, and fatigue cracking [18,19,20,21,22,23]. Furthermore, the influence on mixture performance of different variables, such as size and quantity of the CR, mixing time, binder content, volumetric properties, or the digestion (conditioning) time, has been broadly evaluated [8,17].

As a direct consequence of the technology progress, the use of the dry process to produce CR-modified asphalt mixtures has now reached the potential to be used with confidence in practice. However, unknown details, such as the long-term performance or the recyclability of this kind of asphalt mixtures, must be carefully analyzed. First studies on CR-modified binders reported that they exhibit improved aging resistance in comparison with their corresponding base binder [25,26,27,28,29]. More recently, Xie and Shen [23] experimentally studied the short- and medium-term performance of dense mixtures prepared using the dry process. They analyzed the performance of a Stone Mastic Asphalt (SMA) mixture modified with CR using the dry process after three years in service. By visual inspection, various types of distresses, such as cracking, rutting, raveling, bleeding, pushing, and potholes, were barely found. In addition, some tests conducted on cored samples taken from the field showed slightly higher Marshall stability and lower Marshall flow than control mixtures. Later, they further investigated the aging effect of the storage (conditioning) time on the chemical composition of asphalt binders [30]. They concluded that the CR particles absorbed low molecular weight maltenes, leaving a residual asphalt binder with a higher proportion of asphaltenes. In an earlier study, Rahman et al. [31] had already evaluated the mechanical performance of dense graded asphalt mixtures modified with CR using the dry process after short-term and long-term aging. They concluded that the experimental asphalt mixture modified with CR led to a performance (fatigue and rutting) that was slightly improved following short-term aging, but generally decreased after long-term aging.

The aim of this work was to further the knowledge of the asphalt mixtures modified with CR using the dry process. In particular, the main objective of this study was to assess their aging susceptibility and recyclability. To achieve this goal, a set of SDA mixtures were prepared at laboratory scale by the dry process incorporating two different types of CR. The mechanical performance of these mixtures was evaluated at three aging states, comparing the results with a reference mixture with PmB. This work intends to demonstrate that the addition of CR does not compromise the reuse of the mixture and is comparable to a conventional reclaimed asphalt pavement (RAP) at the end of its service life. To do this, laboratory-prepared RAP was obtained by artificially aging the asphalt mixtures. Then, the aged mixtures, as well as 100% RAP mixtures with rejuvenator, were evaluated, analyzing the differences between the reference PmB mixture and the experimental ones with CR.

## 2. Materials and Methods

**Figure 1:**
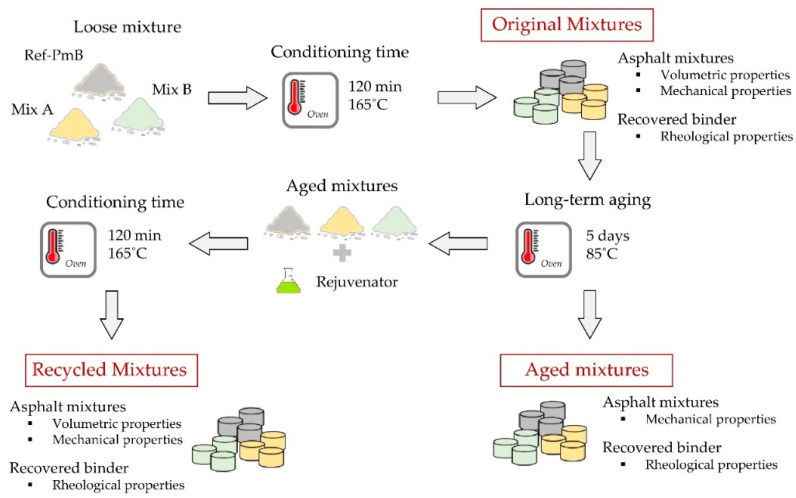
Methodology for the aging and recyclability potential analysis of asphalt mixtures.

In this section, details of the materials and the experimental methods used in the different stages of the study are further described. A scheme with the methodology followed in this study for the analysis of the effect of aging and potential recyclability of asphalt mixtures with CR prepared by the dry process is summarized in Figure 1. 

### 2.1. Asphalt Mixture Preparation and Aging Procedures

**Figure 2:**
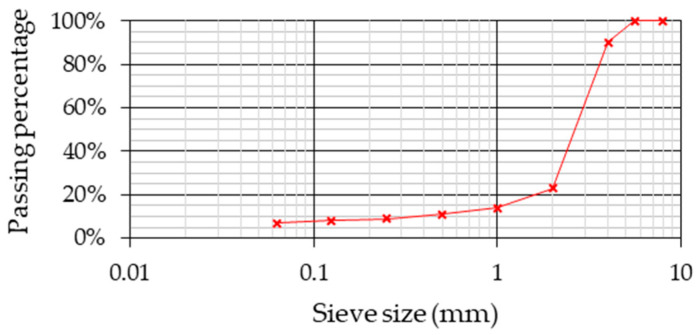
Particle size distribution of the Semi-Dense Asphalt (SDA) mixtures.

Three SDA mixtures with a nominal maximum particle size of 4 mm have been produced: two experimental mixtures modified with two different types of CR using the dry method and a reference mixture. All the mixtures were designed using the same conventional aggregates, resulting in the particle size distribution plotted in Figure 2.

Likewise, specific details of the asphalt mixtures studied are summarized in Table 1. Regarding the asphalt binder type, a conventional 50/70 penetration grade binder was used for the experimental mixtures. According the Swiss standard SNR 640 436:2015, a conventional polymer modified binder (PmB 45/80–65) was used for the reference SDA mixture. The binder content was the same for experimental and reference mixtures (6.2% by weight of mixture). It is noteworthy to mention that it is common to increase the binder content of CR modified mixtures to account for absorption of the binder [8], but this was not the case here.

**Figure 3:**
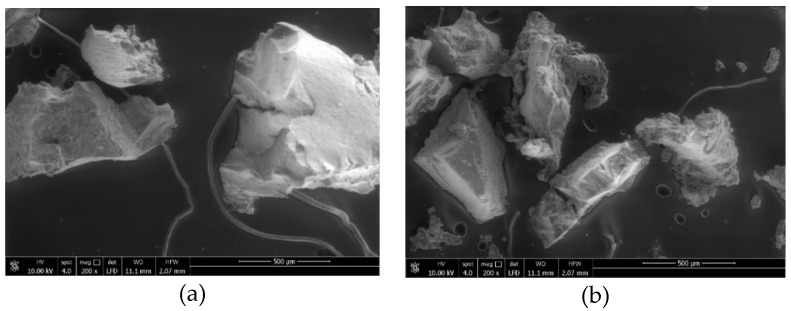
Environmental Scanning Electron Microscope (ESEM) micrographs of the (**a**) first type of crumb rubber (CR) (CR-A) and (**b**) second type of CR (CR-B) particles at 200× magnification.

The only difference between the two experimental mixtures is the type of CR used. The first type of CR (henceforth CR-A) was produced by ambient grinding method and, later, enhanced with a surface treatment using polymers. The second type of CR (henceforth CR-B) was also produced using the ambient grinding, but, in this case, no surface treatment was applied. Both CR types had a maximum particle size of 800 μm. Environmental Scanning Electron Microscope (ESEM) micrographs of each type of particles are shown in Figure 3 [32]. The ESEM experiments were performed with an FEI Quanta 650 by Thermo Fisher (Waltham, MA, USA). As it can be seen in the images, although CR-A particles have been subjected to a polymer coated treatment, there are no clear differences in shape with CR-B particles. However, it seems that the CR-B particles have a more porous and accessible surface. Therefore, any potential benefit as a consequence of the treatment will be related to an optimal chemical activation which would improve the interaction with the asphalt binder.

During the mixing process, the dry method was used to prepare the experimental mixtures. The CR (1 wt.% of mineral aggregate) was directly incorporated into the mixture as additive with no adjustment of the gradation curve or aggregate replacement. First, the preheated aggregates (185 °C) were mixed with the CR (room temperature) for 1.5 min. Then, the preheated binder (160 °C) was added and mixed with the aggregates and CR for another 2 min. The loose mixture was then placed in the oven at 165 °C for 120 min (conditioning time) [33]. Afterwards, cylindrical samples were produced by Marshall compaction at 155 °C.

To simulate the aging effect, an accelerated aging process was carried out in the laboratory. For the CR modified mixtures, the conditioning time was considered enough to simulate short-term aging (associated with the production and installation of the mixture). Likewise, the reference mixture was subjected to the same process (165 °C, 120 min) in order to isolate the influence of the CR of the experimental mixtures during the analysis of the results. Furthermore, to simulate long-term aging (associated with road service life) the Strategic Highway Research Program (SHRP) Long-Term Oven Aging (LTOA) method was applied. This method consists of keeping the compacted specimens in the oven at 85 °C for five days. According to different studies, the application of this method simulates a service life of 7 years for a dry-freeze climate and 15 years for a wet-no-freeze climate [34].

Once tested, and in order to evaluate their recyclability potential, the aged mixtures were considered as artificial RAP. Therefore, 100% RAP mixtures were prepared by using exclusively the artificially aged mixtures. Additionally, commercial Sylvaroad^TM^ RP1000 (Kraton Corporation, Houston, TX, USA) was used as rejuvenating agent in the preparation of a new set of asphalt mixtures. The rejuvenator content was fixed at 5.0% by weight of aged binder. This content was selected in accordance with the experience in previous research works [35]. It is important to note that the recycled CR and PmB modified mixtures were prepared following the same preparation protocol used for the original mixtures. This decision was taken to allow comparison between the mixtures.

### 2.2. Characterization Tests

In order to assess the performance of the different asphalt mixtures, a developed experimental protocol [33] was selected. The laboratory tests carried out were as follows:

- Determination of the volumetric properties of asphalt mixtures: Bulk and maximum densities of several samples were experimentally obtained according to the standards EN 12697-6 (Saturated Surface Dry procedure) and EN 12697-5, respectively. As required, three specimens prepared by impact compactor (50 blows per face) were measured for the original and recycled mixtures. Afterwards, the air void content was calculated for each specimen as the ratio between the bulk and maximum densities as specified in the standard EN 12697-8.
- Marshall test (EN 12697-34): From each mixture and aging state, three different specimens were prepared by impact compactor (50 blows per face). Before testing, the specimens were conditioned in water at 60 °C for 45 min. Thereafter, a load at a constant speed of 50 mm/min was applied to each specimen until failure, recording the deformation obtained throughout the test. This data allowed calculating the corresponding test parameters: stability, flow, and quotient.
- Water sensitivity (EN 12697-12): The Indirect Tensile Strength Ratio (ITSR) is a parameter commonly used to quantify the moisture susceptibility of asphalt mixture. It is defined as the ratio of the Indirect Tensile Strength (ITS) of a water conditioned specimen (wet) and an unconditioned one (dry). In this study, for each asphalt mixture and aging state, a total of six specimens were prepared by impact compactor (35 blows per face) according the specification. The wet samples were conditioned at 40 °C for 72 h. Once conditioned, they were brought to the test temperature (22 °C) in a climate chamber and tested according to EN 12697-23 in order to obtain the corresponding values of ITS.

In addition, asphalt binder samples were recovered from the experimental mixtures at the different aging states in order to analyze the combined effect of CR and rejuvenator on their rheological properties. The asphalt binder was extracted with solvent and recovered by rotatory evaporator (EN 12697-3). Then, the recovered samples were evaluated by means of rheological tests. In particular, complex modulus and phase angle for different test temperatures and frequencies (master curve) were determined [35]. This test was performed following the standard (EN 14770) and using a dynamic shear rheometer (DSR) Physica MCR 301 from Anton Paar (Graz, Austria). These results will be helpful later to understand the mechanisms behind each mixture performance and the role of the different types of CR as elastic particles and binder modifier.

## 3. Results and Discussion

### 3.1. Volumetric Properties of the Asphalt Mixtures

**Figure 4:**
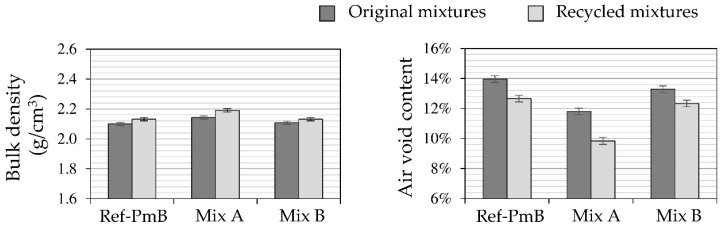
Volumetric properties of the asphalt mixtures.

The volumetric properties of the original and recycled mixtures after the aging processes and addition of the rejuvenator are summarized in Figure 4.

As it can be seen from the results, the type of CR influences the volumetric properties of the asphalt mixture. Comparing to the reference mixture, the CR-modified mixtures show lower air void content, especially the mixture with CR-A. This is expected as the mixture design (gradation curve and/or binder content) was not adjusted to account for the addition of the CR and the consequent swelling process [8]. The added CR particles were partially filling the air voids of the SDA mixtures, reducing their air voids internal structure. Regarding the recycled mixtures, all of them showed a lower air void content than in their corresponding original mixtures. This fact seems to indicate that the incorporation of the rejuvenator (5.0%) does not reverse the action of the aging which finally affects the workability of the mixture.

Concerning the different CR modified mixtures, it was found that the mixture with CR-A shows lower air void content than the mixture with CR-B. Here, the effect of the type of CR can be better observed. As both CR types were produced using the ambient grinding method, they presented a similar shape and size of particles (Figure 3). Nevertheless, the polymer coated treatment applied to CR-A could be the reason leading to a lower air void content. This could have a significant effect on the interaction between asphalt binder and CR particles resulting in changes in the binder viscosity and digestion or swelling levels that justify these differences in the volumetric properties of the asphalt mixtures.

### 3.2. Marshall test

**Figure 5:**
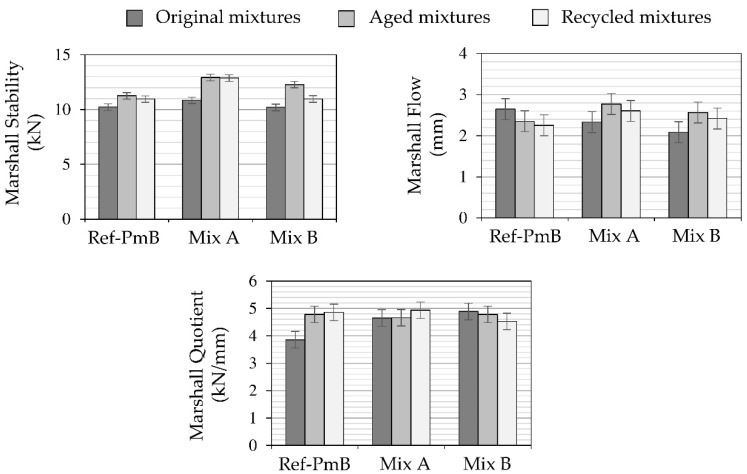
Marshall test results.

Figure 5 shows the Marshall test results for the original, aged (LTOA), and recycled mixtures.

Marshall Quotient (MQ), the ratio of stability to flow, is recognized as an indicator of the resistance of the material to shear stress and is also related to the mixture’s resistance to permanent deformation. High MQ values are associated to high stiffness and better resistance to creep deformation of the asphalt mixture [36].

Comparing to the original mixtures, the experimental CR modified mixtures obtained lower flow values than the ones from reference mixture, finally resulting in higher MQ values. Therefore, these results at high test temperature (60 °C) seem to indicate that CR-modified mixtures are stiffer than the conventional polymer modified asphalt mixture. After aging, the reference mixture reduces its flow and increases its stability, resulting in a higher MQ value. As expected, the mixture is stiffer due to the effect of aging. However, both stability and flow for the CR-modified mixtures increase due to aging, resulting in similar MQ values or even lower in the case of the mixture modified with CR-B. These values reveal a lower aging susceptibility of CR-modified mixtures in comparison with a conventional SDA mixture. This behavior has been previously related to the effect of certain antioxidant compounds used in the tire fabrication process [37]. However, it is highly recommended to validate these results by means of specific tests (e.g., stiffness and fatigue tests).

Comparing the original, aged, and recycled mixtures, it is difficult to draw any conclusions because there are significant differences in air void content. Volumetric properties have a significant influence on the performance of asphalt mixtures. This influence is clearly shown in the MQ values. All the mixtures after recycling process show an MQ value similar to the one obtained for their corresponding aged mixture. In this sense, less air void content leads to stiffer asphalt mixtures [38,39], and it is not possible to isolate the rejuvenator effect in these results.

### 3.3. Water Sensitivity Test

**Figure 6:**
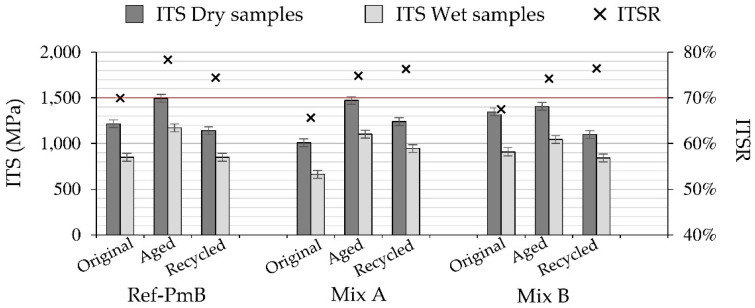
Indirect Tensile Strength (ITS) at 22 °C and water sensitivity test results.

The water sensitivity test results for the original, aged (LTOA), and recycled mixtures are shown in Figure 6.

Analyzing first the overall performance obtained in the original state of the mixtures, the CR-modified mixtures resulted in ITSR values slightly lower to those of the control mixture. In this sense, further work would be required to optimize the CR-modified mixtures in order to meet the requirement established by the Swiss standard (ITSR > 70%). These results could be improved by modifying the mixture recipe or using specific additives to improve the adhesion between binder and aggregates. In terms of ITS, Mix B resulted in higher ITS values than reference and Mix A, indicating higher mixture cohesion [40].

The aging effect can be analyzed by looking at the evolution of the ITS values. After long-term aging, the ITS values increased for all the mixtures. However, this was more significant for the reference mixture, as well as for the Mix A, whereas the Mix B kept its performance and showed only a small change in the strength of the material. These results follow the same trend as the Marshall test results for Mix B, as this mixture showed lower susceptibility to aging than the reference mixture. However, Mix A seems to present a higher susceptibility to aging in this test than in the Marshall test. The effect of aging on ITSR values was similar for all mixtures, increasing this value by around 7% after long-term aging.

As far as recycled mixtures are concerned, all of them showed an adequate performance in this test. The mixtures modified with CR resulted in a slightly higher ITSR value than the reference mixture. In any case, all the mixtures resulted in a good ITSR value and met the requirement established in the standard. As indicated before, these results are only indicative, and it is not possible to directly compare the results obtained in the different aging states (original, aged, and recycled mixtures) due to the differences in the air void content of the mixtures.

### 3.4. Rheological Behaviour of the Extracted Asphalt Binder Samples

**Figure 7:**
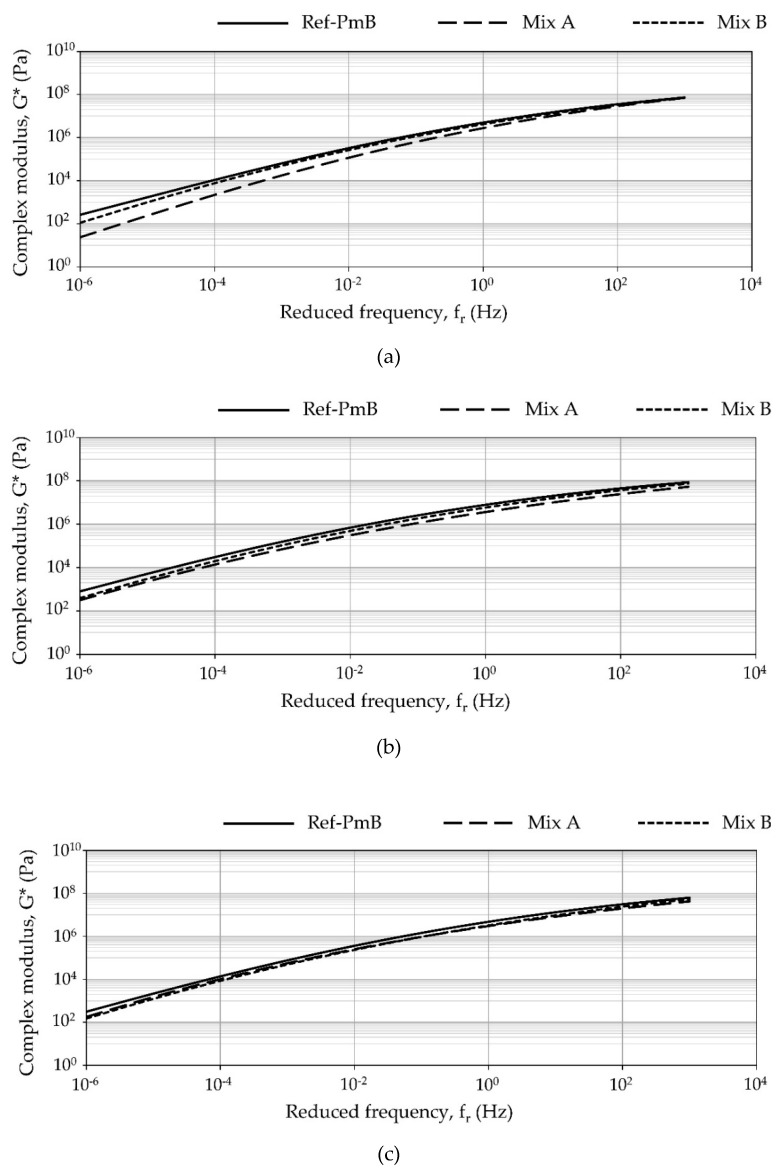
Complex shear modulus (G*) values of the recovered binder from the (**a**) original mixtures, (**b**) aged mixtures, and (**c**) recycled mixtures.

The complex shear modulus (G*) master curves of the extracted binders from the original, aged, and recycled mixtures are shown in Figure 7.

Regarding the original mixtures, at low frequencies (higher temperatures), the polymer modified binder (PmB 45/80–65) used in the reference mixtures is stiffer than the binder samples obtained from the mixtures modified with CR using the dry method. This fact, partially related to the base binder used, could potentially cause some issues for the CR modified mixtures at high temperature. At high frequencies (low temperatures), all binders show similar values of complex shear modulus. Comparison between experimental mixtures shows how the use of untreated CR-B resulted in a higher complex shear modulus of the extracted binder than the use of CR-A, which was originally polymer coated.

As previously discussed, the results obtained from the Marshall tests indicated that the experimental mixtures were stiffer than the reference mixture. The ITS results for the original state indicated that the strength of the experimental mixtures was lower than the reference with CR-B being closer to the reference. The master curves of the recovered binders corroborate the trend seen in the ITS. In any case, it is essential to consider the influence of the CR particles as elastic aggregate on the performance of the mixtures [41] and the effect of test temperature. The Marshal tests are conducted at 60 °C, whereas the ITS characterization is performed at 25 °C. Additionally, the rheological analysis confirmed that the difference in the performance is more prominent at higher temperatures. Therefore, to fully understand the role of the CR particles within the asphalt mixtures, it is highly recommended to validate these results by means of stiffness and fatigue testing. In aged and recycled mixtures, the binder recovered from the original mixture also results in the highest complex modulus values. However, there are fewer differences between the binders than in the original mixtures.

**Figure 8:**
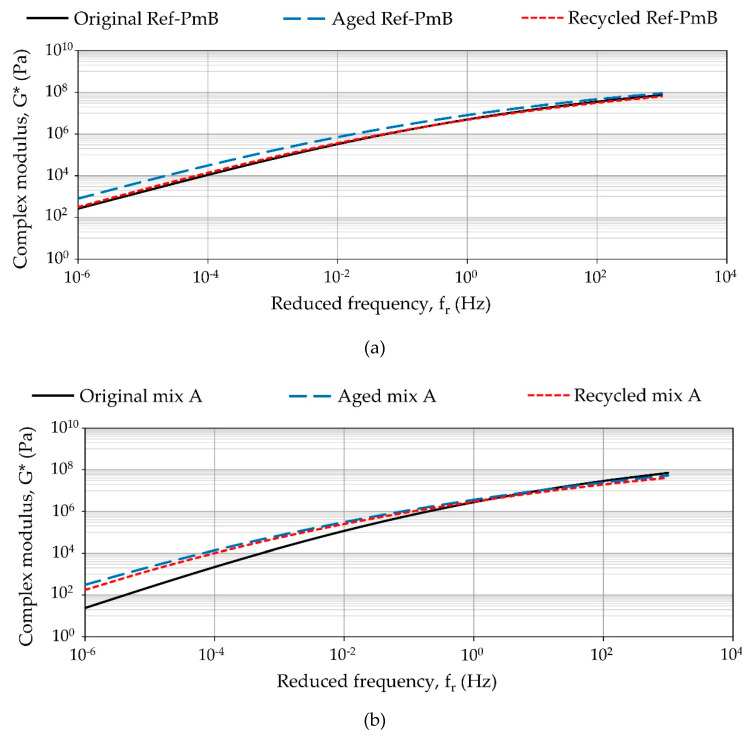

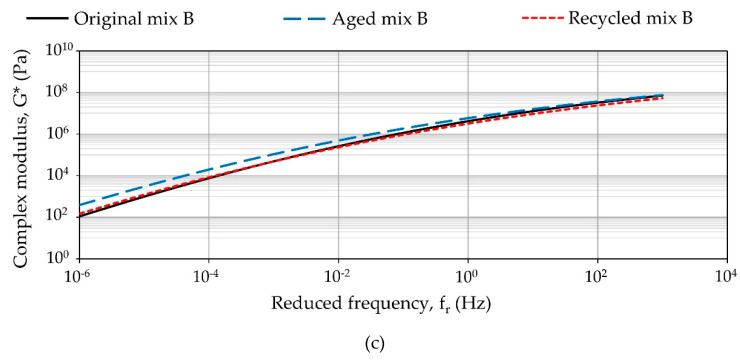
Complex shear modulus (G*) values of the recovered binder from (**a**) reference mixture, (**b**) Mix A, and (**c**) Mix B.

In order to better analyze the difference found in the binders after the long-term aging processes and the effect of the rejuvenating agent on the recyclability for each mixture, the results for the recovered binder samples in the three different conditions are now compared together in Figure 8.

These results indicate that for, all the mixtures, the complex modulus values were increased after long-term aging, especially at low reduced frequencies. The binder recovered from Mix A was found more influenced by the aging process, showing the higher rise in the complex modulus values. Contrary to the Marshall test, where experimental mixtures varied slightly in their results after long-term aging, these results indicate a significant influence of aging on the properties of the recovered binder. This difference highlights that the rheological performances reflect the visco-elastic behavior of binders only. Thus, they are only an indication of possible performance base properties of mixture which not always corroborate the binder results. However, from this study, it can be concluded that antiaging capability and mixture performance of CR modified asphalt binders are enhanced.

Moreover, a reduction in complex modulus for the PmB and the one extracted from Mix B was observed. This confirmed the positive softening effect caused by the use of rejuvenator that resulted in values near those obtained for the original mixtures. Nevertheless, this was not observed for the Mix A. Recently, it was discussed that a potential absorption of the rejuvenating agent by the CR particles could have a side effect on the response [42]. In this case, the addition of rejuvenator in the mixture modified with CR-A only resulted in a less significant reduction of the complex modulus. In this respect, it is difficult to establish a correlation between the results obtained for the mixtures and the results obtained from the binder tests. While the results from the mixtures suggested that both modified mixtures have a better resistance to aging than the reference mixture, the binder analysis confirmed that the type of CR could play a significant role regarding the elastic response added to aged mixtures, as well as the rejuvenator capacity to restore the binder properties to the unaged state.

## 4. Conclusions

In this study, the influence of aging and the recyclability potential of asphalt mixtures modified with CR by the dry process were evaluated. Two SDA mixtures modified with two different types of CR were prepared in the laboratory and compared with a conventional mixture with polymer modified bitumen used as reference. After the analysis of the results obtained from the mechanical and rheological tests, the following conclusions can be drawn:

- In general, the two CR experimental mixtures showed adequate behavior in comparison to the reference mixture with PmB. Nevertheless, further work is required to optimize their design in order to meet the requirements specified in the Swiss standard (ITSR > 70%).
- Regardless the type of CR, the experimental asphalt mixtures were found to be less susceptible to aging than the reference mixture (PmB).
- It was observed that the effect of the CR particles as elastic aggregate was more significant at high temperatures.
- The differences found in the volumetric properties (air void content reduction) and in the rheological response of the binder from recycled mixtures (rejuvenator capability) suggest that the polymer coating changes the resulting performance of the CR negatively.
- Further recommendations include the validation of the experimental results through specific tests (resistance to fatigue and stiffness modulus at different temperatures). Likewise, it will be imperative to take into consideration other parameters at industrial scale (e.g., milling process, transportation, storage, or exhaust gas emissions).

## Figures and Tables

**Table 1:** SDA mixtures composition.

Mixture	Type of Binder	Binder Content	Type of CR	CR Content
Mix A	50/70	6.2%	CR-A	1% by weight of aggregate
Mix B	50/70	6.2%	CR-B	1% by weight of aggregate
Reference	PmB 45/80–65	6.2%	-	-
